# Corrosion, mechanical and bioactivity properties of HA-CNT nanocomposite coating on anodized Ti6Al4V alloy

**DOI:** 10.1007/s10856-022-06655-6

**Published:** 2022-03-26

**Authors:** Faezeh Dalili, Rouhollah Mehdinavaz Aghdam, Reza Soltani, Mohsen Saremi

**Affiliations:** School of Metallurgy and Materials Engineering, College of Engineering, University of Tehran, P.O. Box: 11155-4563, Tehran, Iran

## Abstract

Hydroxyapatite-carbon nanotubes (HA-CNTs) nanocomposite coating was applied by electrophoretic method on anodized Ti alloy to investigate its stability in simulated body fluid (SBF). The biocoating was characterized by using scanning electron microscope (SEM) for microstructure, X-ray diffraction (XRD) for crystallography. The effect of CNTs concentration on the coating properties was also investigated and found out that CNTs up to 5% has various improving effect on the system. It increased corrosion resistance and adhesion of the coating to the substrate and decreased the number of cracks on the coating. The results of the in vitro test showed that the cell viability increased with increasing the concentration of CNTs to 3 wt.% CNTs.

**Figure:**
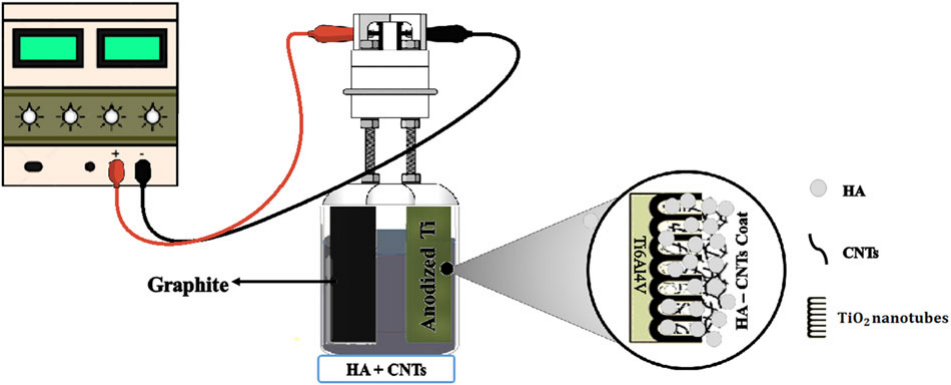
Graphical abstract

Graphical abstract

## Introduction

Extensive research is being done on the bio implant materials due to the increasing need to alleviate the damage caused by aging, disease and accidents. Titanium and its alloys are used as implants in biomedical applications due to their excellent corrosion resistance, high strength, low density and elastic modulus [1]. The bioactivity of the metallic implants is an important issue which may limit their application. One approache used to modify the surface of titanium implants for bioactivity is to coat the titanium with HA [2, 3]. HA coatings have been shown to increase biocompatibility and bioactivity extensively due to its biological and chemical similarity to bone and its direct bonding to bone without the formation of fibrous capsules [4, 5].

However, HA has disadvantages such as brittleness, low tensile strength and fracture toughness [6–8] where used in load bearing points and it is challenging to create a coating without crack [9, 10]. In this regard, HA-based composite coatings have been developed [11–16]. Many materials have been used for composite with Ha including; CNT, GO, Al2O3, ZrO2 etc….. but among the various materials used as reinforcements in hydroxyapatite coatings, reinforcing HA coatings with CNT is a promising solution with more desirable properties such as higher fracture toughness, elastic modulus, hardness and shear strength than single-layer HA coating [14, 16, 17]. In research by Khazeni et al. [18], it was observed that the presence of CNTs in composite coatings containing HA bridges between hydroxyapatite structures and fills empty spaces in the coating to improve its mechanical properties. According to research by Askari et al. [19], HA/YSZ/Al2O3 coatings were more bioactive and more resistant to corrosion than HA coating. Ming et al. [13] deposited hydroxyapatite and graphene oxide coatings on the titanium surface by electrophoretic deposition. The results showed a reduction in surface cracks, increased adhesion strength, and improved coating corrosion behavior by adding GO and CNT to HA coatings.

Another approach used to modify the titanium was the anodizing process, before applying the HA coating, to improve the adhesion strength of HA coating to the titanium surface [20]. The formation of a layer of titanium oxide nanotubes on the surface of titanium by anodizing process increases the bond strength between the HA layer and the Ti substrate [20].

Thermal spray is usually used to apply HA composite coatings which may bring problems such as thermal decomposition of hydroxyapatite, heterogeneity in structure, changes in substructure, surface morphology and composition [21].

One of the promising methods for deposition of HA coatings is the use of electrophoretic method, which does not follow the mentioned problems and have advantages such as simplicity, low-cost equipment, ability to control coating microstructure, ability to form a coating of uniform thickness, the ability of coating complex-shaped substrates and fast coating rate [22]. In the best of our knowledge no electrophoretic deposition of HA-CNT composite coating have been done on Ti anodized nanotubes. The aim of this work is to apply HA/CNT composite coating on anodized Ti surface nanotubes by electrophoretic method and to study its corrosion,bioactivity and mechanical properties.

## Experimentals

### Preparation of substrate

Samples were cut from Ti6Al4V sheet (ASTM 265-10, LOTERIOS Co.) dimensions of 2 × 15 × 2 mm^3^ as a substrate, polished to mirror surface with SiC paper to 2500 grits and then cleaned ultrasonically in acetone alcohol mixture and rinsed in ditilled water. Then, the samples were chemically cleaned in acidic solution (HF: HNO_3_:H_2_O = 1:4:5) for 15 min and immediately washed with distilled water and dried in air.

### Formation of TiO_2_ nanotubes by the anodizing process

An electrolyte solution containing 0.5 wt.% ammonium fluoride (NH_4_F, Merck), 10 wt.% distilled water and ethylene glycol (C_2_H_6_O_2_, Merck) was prepared. For anodizing, Ti6Al4V samples as an anode electrode and graphite as the cathode electrode were connected to the positive and negative poles of the power supply, respectively. Anodizing was performed at 60 V for 60 min. After anodizing, the samples were annealed in the furnace at 450 °C to for 2 h to form anatase structure.

### Electrophoretic deposition of HA-CNT coating

To prepare the suspension, 0.25 g of HA (Sigma-Aldrich) was added to 50 ml of 2-propanol (C_3_H_8_O, Merck) and stirred magnetically for 3 h and ultrasonically for 15 min. Suspensions with CNTs concentrations of; 0, 2, 3 and 5 wt.% of -COOH functionalized MWCNTs powder (5–15 nm diameter and 50 μm length, US research nanomaterial) were prepared. and labeled as HA, 2CNT/HA, 3CNT/HA and 5CNT/HA, respectively. The specified amounts were added to the above suspension and stirred magnetically and ultrasonically for 3 h and 15 min. Finally, the pH of the solution was adjusted to 3 with nitric acid (HNO_3_, Merck). Two-electrode cell systems, including anodized sample as cathode and graphite as anode, were used for EPD process and was performed under 10 V for 8 min. The samples were then sintered for one hour at 800 °C in a furnace under argon atmosphere.

### Characterization of coatings

The surface morphology and analysis of the samples were observed by Scanning Electron Microscope (SEM; VEGA III LMU, FESEM; MIRA III, TESCAN) equipped with energy dispersive X-ray (EDX) attachment.

The phase analysis of the samples was performed by X-ray diffraction (XRD, X’ Pert Pro, Phillips) method. The diffraction patterns were obtained using K_αcu_ radiation (λ = 1.54 Å, 40 kV, 30 mA) in the range of 5° < 2θ < 100°, step size 0.02°.

### Hardness and adhesion strength tests

The adhesion strength of the coatings was measured according to ASTM D3359 and the degree of adhesion of coating was classified on a 0–5B scale. While 5B and 0B show the best and worst adherent coating, respectively. The hardness of the coated samples was determined using the Vickers Micro-hardness (V-Test II, BARESISS) test. A load of 3 N was applied for a dwell time of 10 s. An average of three measurements is reported for each sample.

### Study of corrosion behavior

To study the corrosion behavior of the coatings, a conventional three-electrode electrochemical cell was used consisting of a saturated calomel electrode (SCE) as reference, a platinum wire as counter electrode and the samples as a working electrode in simulated body fluid (SBF) at 37 ± 2 °C (pH=7.42). The SBF was prepared according to the Kokobo et al. method [23]. The electrochemical impedance spectroscopy was done by using EG&G 273 electrochemical stystem, applied in the frequency range of 100 kHz to 10 mHz with a sinusoidal voltage of 10 mV. Potentiodynamic polarization was performed according to ASTM G5 standard at a scan rate of 2 mV/s. All EIS data were interpreted using ZSimpWin software.

### In vitro biocompatibility studies

#### Bioactivity evaluation

An immersion test in SBF solution was used to evaluate the coatings’ ability to form apatite on the coating surface. Calculate the volume of SBF that is used for testing using the following equation [23]:

1 $${{{\mathrm{V}}}}_{{{\mathrm{s}}}} = \frac{{{{{\mathrm{S}}}}_{{{\mathrm{a}}}}}}{{10}}$$

where V_s_ is the volume of SBF (ml) and S_a_ is the apparent surface area of the sample (mm^2^). For this purpose, the samples were immersed in SBF solution (pH=7.42) at 37 ± 2 °C for 14 days. SEM with EDX was used to observe and investigate apatite formation on the surface of samples.

#### Cell culture

MG63 Cell line (Pasteur Institute, Iran) were used in all sections of cell culture. The MG63 cells were cultured in culture medium (Dulbecco’s minimal essential medium (DMEM), 10% (v/v) fetal bovine serum (FBS) and 1 (v/v)% streptomycin/penicillin) at 37 °C in a humidified atmosphere containing 5% CO_2_. To sterilize the samples, the samples were placed in 70% ethanol for 30 min. After washing three times with saline phosphate buffer solution (PBS), they were exposed to UV light for 30 min. The samples were placed in 24-well cell culture plates and under sterile conditions. In each well containing the sample, 2 ml of culture medium was added. Then 10^4^ cells/well were seeded on samples and incubated in a humidified atmosphere with 5% CO_2_ at 37 °C.

#### Cell viability study

3-(4,5-dimethylthiazole-2-yl)-2,5-diphenyl tetrazolium bromide (MTT) assay was employed to evaluate the viability and proliferation of MG63 cells on the surface of samples. After 24 and 72 h incubation, the culture medium was removed.150 μl of MTT solution (5 mg/ml in a PBS) was poured into each well and incubated for 4 h in a humidified atmosphere with 5% CO_2_ at 37 C. dindimethyl sulfoxide (DMSO) was used to dissolve the formazan crystals. Then the purple solution was transferred to a new 96-well cell culture plate and its optical density (OD) was measured at the wavelength 0 f 570 nm using a microplate reader (STAT FAX 2100, USA) against DMSO (blank). Finally, the relative cell viability was expressed as following [24, 25]:

2 $$Cell\,viability\,\% = \frac{{A_{Sample} - A_{Blank}}}{{A_{Control} - A_{Blank}}} \times 100$$

where A_Sample_, A_Blank_ and A_Control_ were the absorbances of the sample, blank (DMSO) and tissue culture plate (TCP, control), respectively.

#### Cell morphology study

After 24 h of culture, the samples were then washed with phosphate buffer solution (PBS) and fixed with 4% paraformaldehyde for 30 min. Samples were dehydrated in the graded concentrations of ethanol (30, 70, 90, 96 and 100 (v/v) %) for 10 min, respectively. After complete drying at room temperature, the morphology of the fixed cells on the samples was examined by SEM.

## Results & discussion

### Characterization of anodized titanium oxide

**Fig. 1:**
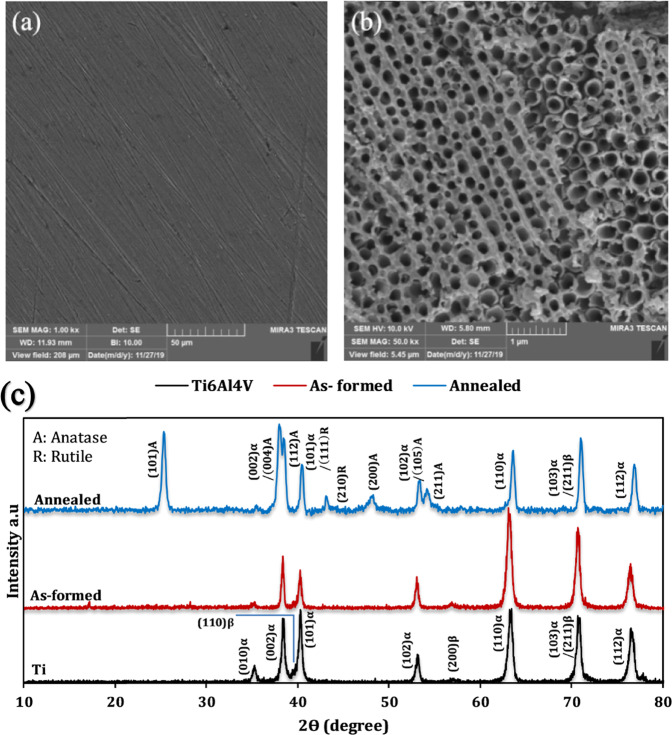
FESEM image of surface (**a**) Ti6Al4V sheet (**b**) Anodized Ti6Al4V sheet at 60 V for 1 h. **c** XRD spectra of titanium and anodized titanium samples

Figure 1a shows the FESEM image of the pre-anodized sample surface and Fig. 1b shows the surface after anodizing process. During the anodizing at the cathode, water decomposes to produce dihydrogen gas [26]:

3 $$2{{{\mathrm{H}}}}^ + + 2{{{\mathrm{e}}}}^ - \to {{{\mathrm{H}}}}_2$$

At the anode, titanium oxidation (Eq. (4)) and water decomposition occur and produce dioxygen gas (Eq. (5)):

4 $${{{\mathrm{Ti}}}} \to {{{\mathrm{Ti}}}}^{4 + } + 4{{{\mathrm{e}}}}^ -$$

5 $$2{{{\mathrm{H}}}}_2{{{\mathrm{O}}}} \to {{{\mathrm{O}}}}_2 + 4{{{\mathrm{H}}}}^ + + 4{{{\mathrm{e}}}}^ -$$

To form the oxide layer, the Ti^4+^ ions react with the O^2−^ originating from water decomposition (Eq. (6)). In fluoride-containing electrolytes, the presence of fluoride ions causes the formation of water-soluble species [TiF_6_]^2−^ on the substrate (Eq. (7)). Fluoride ions move rapidly to accumulate toward the metal-oxide interface and grain boundaries [27].

6 $${{{\mathrm{Ti}}}} + 2{{{\mathrm{H}}}}_2{{{\mathrm{O}}}} \to {{{\mathrm{TiO}}}}_2 + 4{{{\mathrm{H}}}}^ + + 4{{{\mathrm{e}}}}^ -$$

7 $${{{\mathrm{TiO}}}}_2 + 6{{{\mathrm{F}}}}^ - + 4{{{\mathrm{H}}}}^ + \to \left[ {{{{\mathrm{TiF}}}}_6} \right]^{2 - } + 2{{{\mathrm{H}}}}_2{{{\mathrm{O}}}}$$

The oxide layer is chemically dissolved by fluoride ions to form corroded cavities on the surface. Therefore, the proper balance between the two reactions of oxide formation and chemical dissolution can create a uniform array of TiO_2_ nanotubes by the anodizing method [28].

Titanium oxide nanotubes with an average pore diameter of about 150 nm and a wall thickness of about 23 nm were formed on the titanium surface. The dimensions of TiO_2_ nanotubes, such as their diameter and length, have deep effects on the properties of the coatings deposited on them. The dimensions of TiO_2_ nanotubes should be proportional to the size of the particles deposited on them as coatings so that the particles can easily penetrate the tubes and the gaps between the nanotubes. Thus, a strong mechanical interlocking is created between the coating particles and the nanotubes, which increases the mechanical bond between the coating and the substrates [29–32]. For example, according to research by Zhang et al. [29], the bond strength of HA coating on TiO_2_ nanotubes increased from 8.87 to 28.7 MPa with an increasing nanotube diameter from 23.4 to 136.8 nm. In most studies, nanotubes with a length in the micrometer range were synthesized in electrolytes based on ethylene glycol and ammonium fluoride [28, 33]. For example, Kim et al. [34], in almost the same conditions of our study except for water content in the electrolyte (2.2% vol.%) synthesized TiO_2_ nanotubes with an approximate length of 14.6 μm. Also, they showed that with increasing water content, the nanotubes have a shorter length [34]. In the research of Qadir et al. [35], anodizing was performed at 30 volts and other similar conditions of our research and TiO_2_ nanotubes with a length of 0.88 μm were synthesized. In addition, the length of nanotubes depends on the voltage and is expected to increase with increasing voltage [29, 34, 36]. Therefore, the length of the TiO_2_ nanotubes synthesized in the present study is estimated to be about 0.88 μm<*l*<15 μm.

Figure 1c shows the XRD results for Ti6Al4V, anodized titanium (As-formed), and annealed samples. The XRD spectrum of Ti6Al4V alloy, due to its two-phase nature, shows the peaks according to the JCPDS card standard with numbers 01-089-5009 and 01-077-3482 for phase α and β, respectively. In the XRD spectrum of the anodized sample, only the characteristic peaks of titanium are visible. The reason for the absence of TiO_2_ peaks in the anodized sample spectrum are the amorphous nature of TiO_2_. By annealing at 450 °C. The amorphous nanotubes became an anatase structure and showed peaks according to the standard anatase pattern numbered 00-021-1272. Also, some weak rutile peaks according to the standard number 00-021-1276 have appeared in the XRD spectrum of the anodized sample.

### Characterization of the coatings

**Fig. 2:**
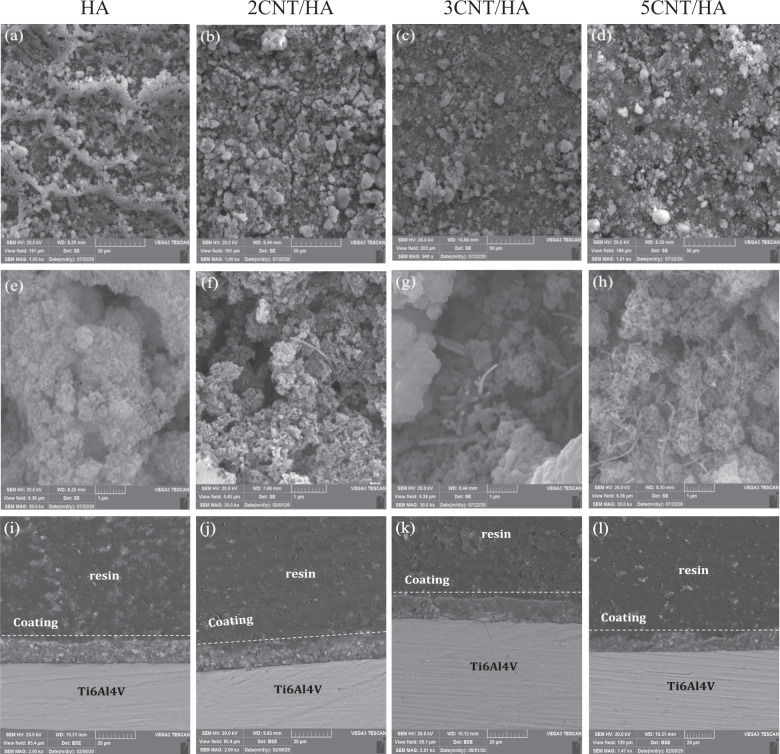
SEM images of the surface coatings: top at low magnification and middle at high magnification and bottom SEM images of the cross-section of coatings: (**a**, **e**, **i**) HA, (**b**, **f**, **j**) 2CNT/HA, (**c**, **g**, **k**) 3CNT/HA, (**d**, **h**, **l**) 5CNT/HA

Figure 2 shows the SEM images of the surface morphology and cross section of HA-CNT composite coating on Ti. It can be seen from SEM images in Fig. 2b-d that the surface cracks decreased with increasing CNTs concentration from 0 to 5 wt.%. Some cracks are seen on the composite coatings which may be due to the solution evaporation and/or residual stress during thick coating formation. The difference in thermal expansion betwwen the substrate and thick coating can also lead to crack formation and expansion.

Figure 2e–h shows that CNTs try to reduce the interfacial microcracks in the coating microstructure and increase the bond strength between the particles through the bridging mechanism in the cracks and prevent the breakage of the bond [18]. It can be concluded that the addition of CNT increases the coating density, removes the agglomerates, and reduces the cracking.

Similar to the report [37], the SEM images in Fig. 2 show the presence of micro-pores on the surface of all composite coatings which is due to the evaporation of excess water and propanol during drying. Micro-pits are generally useful for the adhesion of cells and tissues because they provide sites for cell attachment and cells adhere better to the surface of the micro pits [38, 39]. According to the cross-section images in Fig. 2i-l, good coating quality can be observed in terms of uniformity and adhesion of the coating to the substrate surface and thickness of the coating. The thickness of HA, 2CNT/HA, 3CNT/HA and 5CNT/HA are 10, 9, 8 and 7 µm, respectively. As the concentration of CNTs increases, the coating thickness decreases from 10 to 6 μm, indicating the role of CNTs in increasing the viscosity of the suspension. It, therefore, results in slower movement of HA-CNTs composite nanoparticles and less deposition on the substrate [25].

**Fig. 3:**
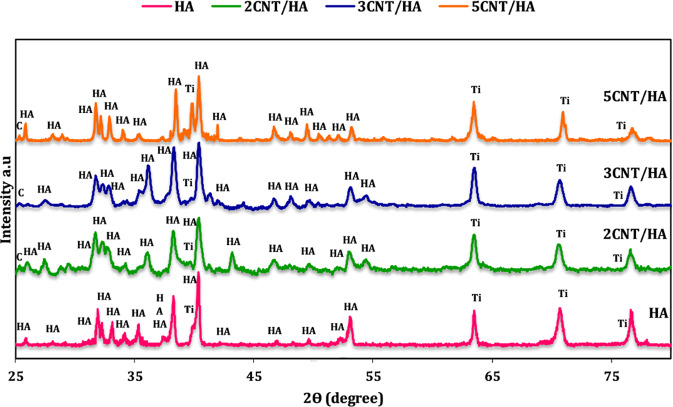
XRD spectrum of coated samples of HA with different concentrations of CNTs

To investigate the crystal phase of the coating XRD test were performed and the results are shown in Fig. 3 Comparing the obtained peaks with standard JCPDS cards showed that there is no calcium phosphate phase other than HA in the structure. The presence of sharp peaks with the lowest level of HA flattening indicates high crystallinity and stoichiometry. In addition to peaks related to HA, peaks related to anodized titanium substrate can also be detected A weak peak for CNTs at an approximate position of 2θ = 26 ° is seen in this pattern.

### Evaluation of mechanical behavior of the coatings

#### The adhesion strength of coatings

**Fig. 4:**
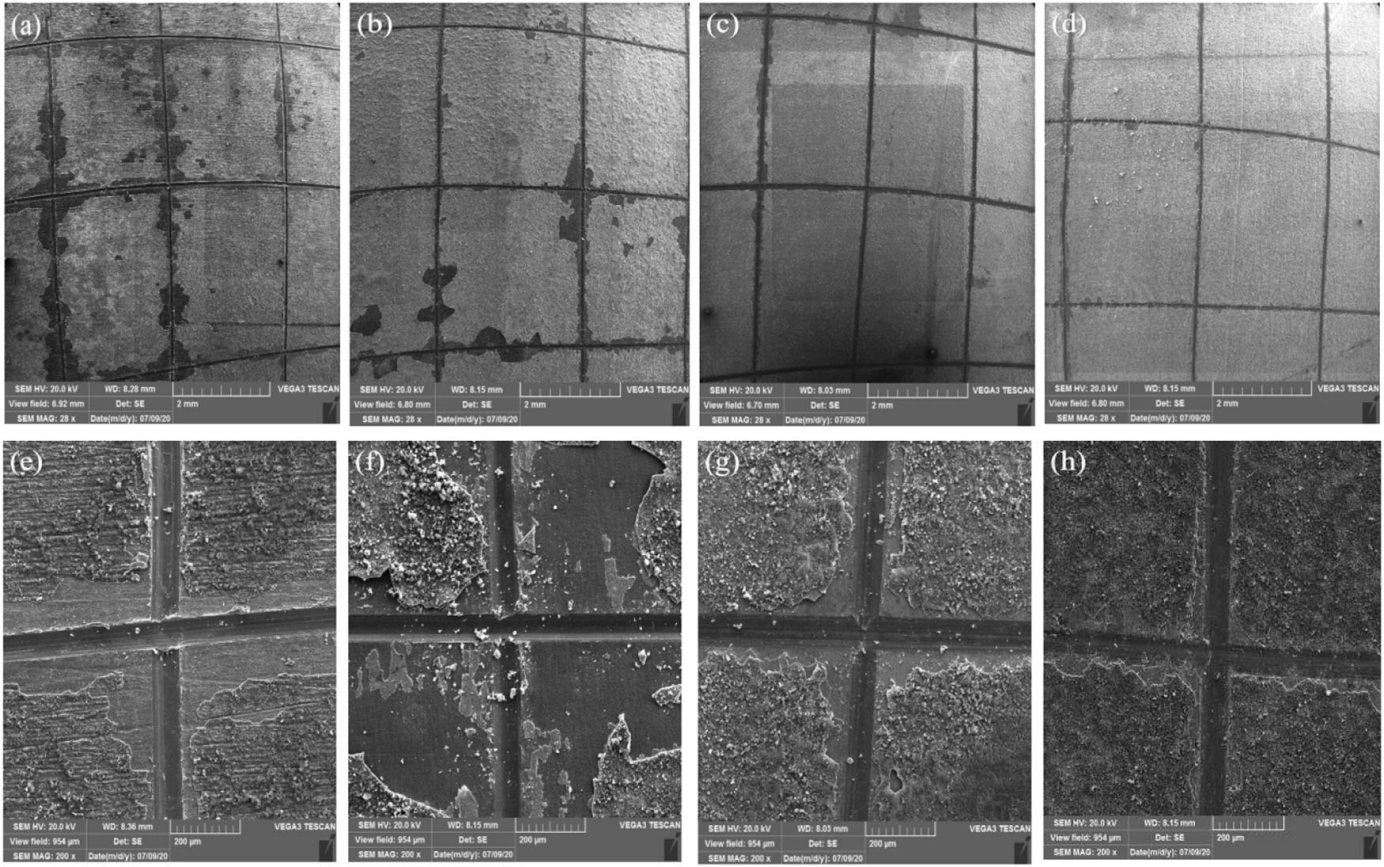
SEM images of the surface of samples covered by the electrophoretic method after cross Cut test: top with low magnification and down with high magnification: (**a**, **b**) HA, (**c**, **d**) 2CNT/HA, (**e**, **f**) 3CNT/HA, (**g**, **h**) 5CNT/HA

SEM images of the surface of samples coated with different concentrations of CNTs after the Cross Cut adhesion test are shown in Fig. 4. But the presence of CNTs as a reinforcing phase creates a dense and uniform coating with positive effect on the adhesion of the coating to the substrate and cohesion of the particles to each other. The adhesion between particles occurs by creating an entangled structure through the bridging mechanism between HA particles. The results of the coating adhesion classification, which was evaluated by the Cross Cut adhesion test, are shown in Table 1.

**Table 1:** Coating adhesion classification results

Samples	Coating adhesion classification 0-5B	Hardness (HV)
HA	2B	457
2CNT/HA	2B	458
3CNT/HA	3B	466
5CNT/HA	4B	553

#### The microhardness measurement of the coatings

The results of the microhardness test are reported in Table 1 in which it is shown that with the addition of CNTs, the hardness of the coating has increased. Such improvement in hardness is due to the strengthening of matrix and grain size refinement, preventing plastic deformation [40]. These results are similar to the results obtained from other studies [14, 16, 41]. Increasing the microhardness of HA coating containing CNT can improve their wear resistance and thus increase the life time of orthopedic implants.

### Investigation of corrosion behavior of coatings

#### Open circuit potential test

**Fig. 5:**
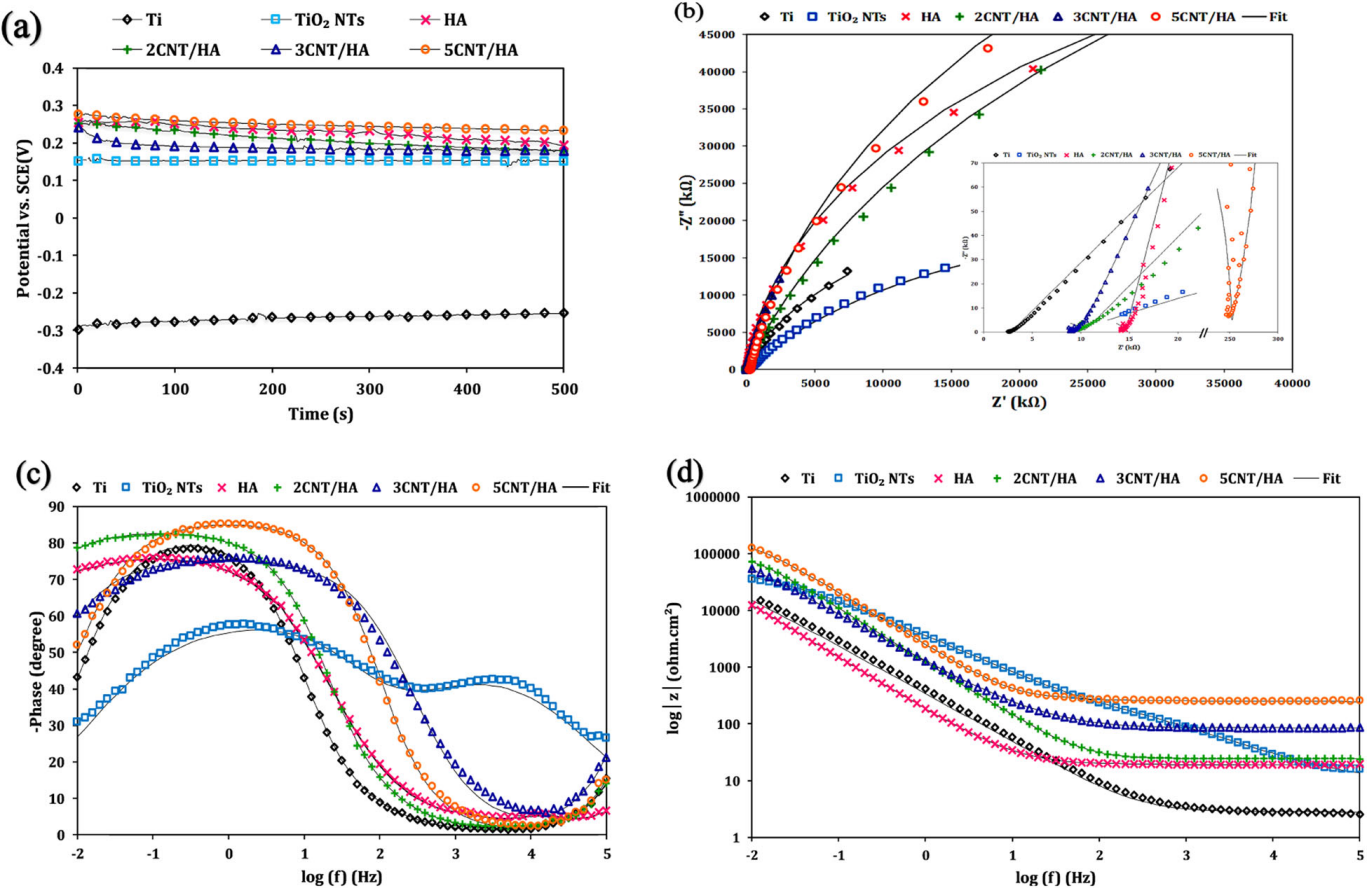
**a** Open circuit potential diagram titanium and coated samples, (**b**) The Nyquist curve of uncoated and coated titanium samples, (**c**) bode-phase curve, (**d**) bode-impedance curve

The Open Circuit Potential (OCP) diagrams of the samples are shown in Fig. 5a. In general, the OCP of uncoated titanium samples has increased slightly over time towards noble potentials. This relative increase is probably related to the formation of oxides or corrosion proddue on the sample surface. The slight fluctuations seen in open circuit potential curves may be due to local corrosion in some parts, including coating defects. The OCP value of uncoated titanium is −0.25 V vs. SCE. While the OCP values of HA, 2CNT/HA, 3CNT/HA and 5CNT/HA samples were 0.15, 0.19, 0.18 and 0.23 V vs. SCE, respectively. The OCP value for all coated samples shifted significantly in the noble direction, indicating higher thermodynamic stability by addition of CNT to HA coating. The reinforcing phase of CNT makes the structure more compact by bridging between HA particles and makes it more difficult for the solution to penetrate to the surface.

#### Electrochemical impedance studies

The results of the electrochemical impedance test in the form of Nyquist diagrams for uncoated and coated samples in SBF solution are shown in Fig. 5b. The small semicircle in high-frequency in the Nyquist diagram corresponds to the electrical behavior of the coating. The large semicircle in the low-frequency in the Nyquist diagram relates to the electrical behavior of the titanium surface, which deals with adsorbed species and electrochemical reactions at the titanium surface.

The bode-phase diagram of the samples is shown in Fig. 5c. The reduction of the phase angle of the samples in the high-frequency zone may be related to the porous nature of the outer layer. In the middle frequency range, the phase angle increases to about 80 and is relatively constant up to low frequencies, indicating passive thin oxide on the surface and passive oxide having a behavior close to the capacitor. In the low-frequency region, the phase angle is decreased due to passive oxide resistance [42, 43]. All samples showed a maximum phase angle in the range of 70–85° over a wide range of frequencies (low frequency to medium frequency). The flattening of the wind-phase diagram of the coatings at an angle of about 70 indicates the passive behavior of the coatings, which prevents corrosion. Porous TiO_2_ nanotubes, a maximum of two phases appears in the high and low-frequency region. They can be attributed to the bilayer structure of TiO_2_ nanotubes, with an inner dense oxide barrier and an outer porous film formed on the surface.

The bode -impedance curve is shown in Fig. 5d. As the concentration of CNT increases, the absolute impedance value increases. Higher impedance values indicate an increase in corrosion resistance. Higher impedance indicates the presence of passive protective films on the titanium alloy.

**Fig. 6:**
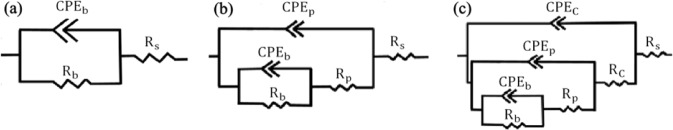
Equivalent circuit: (**a**) titanium sample, (**b**) anodized titanium and (**c**) coated samples

To deduce quantity values of EIS data, an equivalent circuit was used, as shown in Fig. 6. The electrochemical response for ceramic coatings is not considered a pure capacitor but is replaced by a fixed phase element (CPE) in the circuit due to the presence of porosity, heterogeneity and possibly cracks on the coating surface. Each time constant consists of a resistor (R) and a constant phase element (CPE). The CPE impedance equation is defined as follows:

8 $${{{\mathrm{Z}}}}_{{{{\mathrm{CPE}}}}} = \frac{1}{{\left[ {{{{\mathrm{Y}}}}_{{{\mathrm{o}}}}\left( {{{{\mathrm{j}}}}\omega } \right)^{{{\mathrm{n}}}}} \right]}}$$

where, Y_°_ is equal to the CPE constant, j imaginary unit ($\sqrt 1$), ω equal to the angular frequency in rad⁄s and n is an experimental power whose value is between zero and one. A value of n equal to zero indicates a net resistance and a value of one indicates a pure capacitor.

The titanium sample fits a modified rendering circuit consisting of a solution resistance element (R_s_), a constant phase element associated with a barrier oxide layer (CPE_b_) and a barrier oxide layer resistance element (R_b_), as is shown in Fig. 6a. R_b_ corresponds to the electric double layer in the metal-coating interface that result from the interaction of the attacking ions and the metal substrate.This circuit model has already been used to describe non-anodized titanium substrates [44]. The equivalent circuit used for anodized titanium, consisting of two oxide layers with two-time constants, namely an inner barrier oxide layer and an outer porous oxide layer, and is consistent with the equivalent circuit proposed in previous work [42]. R_p_ and CPE_b_are the resistance and the constant phase element of the porous oxide, respectively. R_C_ and CPE_C_ show the resistance and the constant phase element of the electrophoretic coating, respectively. R_C_ shows the resistance of the coating to the transfer of corrosive species from the solution to the substrate and the transfer of metal ions to the corrosive medium. The values of R_s_ for each sample is a function of the SBF electrolyte solution and the distance between the reference and the work electrodes in the electrochemical cell.

According to the values in Table 2, it can be concluded that the corrosion protection of the samples is dominated by R_b_. This indicates that the high corrosion performance of samples is almost dictated by the strength of their barrier layer. In fact, with increasing concentration of CNTs, the adhesion of the coating to the substrate increases and leads to reduces the presence of adsorbed species and electrochemical reactions on the Ti surface. Coating surface roughness depends on the amount of experimental power of the constant phase element of the coating and as it decreases, the surface roughness increases. According to Table 2, the value of n for anodized titanium is 0.59. Due to the porous nature of anodized titanium, the n parameter decreased from 0.84 for titanium to 0.59, indicating an increase in surface roughness. Also, the value of n corresponds to the linear slope of the bode-impedance diagram. It is quite clear that when n is close to 1, the surface is uniform and smooth. On the other hand, low values of n indicate the deviation from the ideal capacitive behavior, attributed to the heterogeneity of the surface [45].

**Table 2:** Values for the different elements of the electric equivalent circuits whose response fitted the data obtained for uncoated and coated titanium samples

Samples	R_s_ (Ω.cm^2^)	CPEc (μF.cm^−2^)	n_c_	R_c_ (Ω.cm^2^)	CPEp (μF.cm^−2^)	n_p_	R_p_ (kΩ.cm^2^)	CPE_b_ (μF.cm^−2^)	n_b_	R_b_ (kΩ.cm^2^)
Ti	2.80	–	–	–	–	–	–	620.60	0.84	51.26
TiO_2_NTs	9.00	–	–	–	549.10	0.59	0.36	276.70	0.74	61.60
HA	9.18	0.28	1	14.50	135.70	0.96	1.06	225.30	0.23	101.55
2CNT/HA	10.25	0.15	1	11.26	80.90	0.66	16.50	82.90	0.92	160.43
3CNT/HA	9.88	0.06	1	166.20	65.40	0.68	21.02	78.55	0.90	172.78
5CNT/HA	9.94	0.01	1	251.70	30.30	0.74	27.04	73.30	0.92	185.40

#### Polarization

**Fig. 7:**
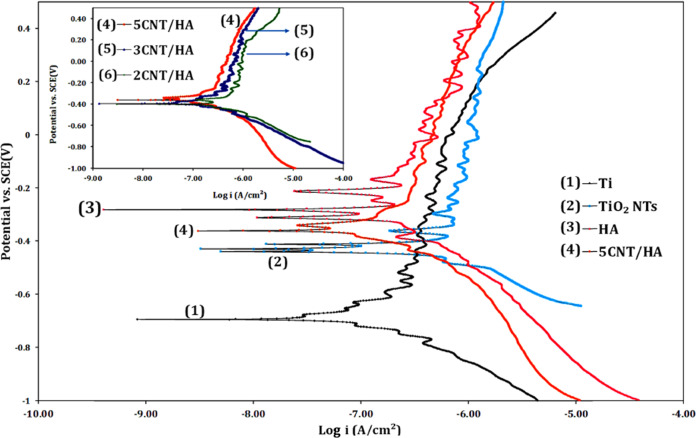
Polarization curve of uncoated and coated titanium samples

The polarization curves of the various samples are shown in Fig. 7. The values of the polarization parameters obtained from the diagrams are presented in Table 3. According to the polarization diagrams, the amount of E_corr_ titanium coated with HA is −0.29 vs. SCE, indicating a change in potential in a noble direction compared to the uncoated titanium sample (E_corr_ = −0.69 V vs. SCE). E_corr_ values for 2CNT/HA, 3CNT/HA and 5CNT/HA samples were −0.40, −0.36 and −0.34 vs. SCE, re spectively. i_corr_ values for HA, 2CNT/HA, 3CNT/HA and 5CNT/HA samples were 0.11, 0.18, 0.15 and, 0.14 μA/cm^2^ respectively. Coatings containing CNTs have a higher i_corr_ compared to HA single-component coatings. This means that by adding CNTs to the coating, the corrosion resistance is reduced compared to the one-component HA coating. This behavior is due to the increase in micro-pores around the CNT. After water loss and drying of the coating, many micro-pores are created, so the electrolyte infiltrates and enters the coating structure. Therefore, due to the penetration of the electrolyte in the structure of coatings containing nanotubes, corrosion resistance is reduced. But complex CNTs net prevent the mobility of ions and electrolyte molecules present in the micro-pores of CNTs net and therefore do not affect the conductivity of the coating [46].

**Table 3:** Parameters obtained from the polarization curve of uncoated and coated titanium samples

Samples	E_corr_(V vs. SCE)	I_Corr_(μA/cm^2^)
Ti	−0.69	0.17
TiO_2_NTs	−0.42	0.22
HA	−0.29	0.11
2CNT/HA	−0.40	0.18
3CNT/HA	−0.36	0.15
5CNT/HA	−0.34	0.14

### Biocompatibility properties

#### Bioactive behavior of coatings by immersion in SBF solution

**Fig. 8:**
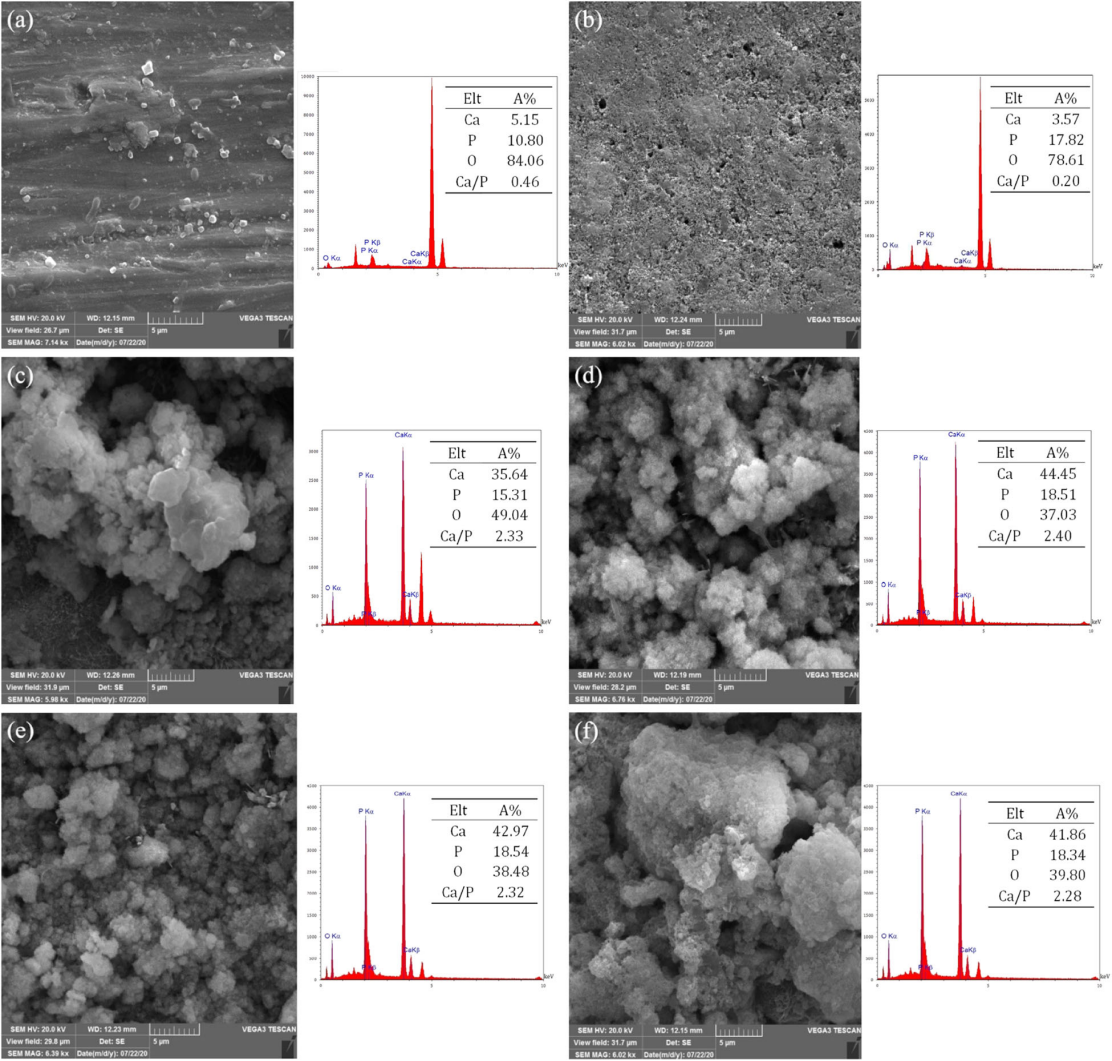
SEM images of the samples surface after immersion for 14 days in SBF solution with EDX spectrum: (**a**) titanium, (**b**) anodized titanium, (**c**) HA, (**d**) 2CNT/HA, (**e**) 3CNT/HA and (**f**) 5CNT/HA coatings

The coated samples were immersed in SBF solution at 37 °C for 14 days. According to Fig. 8, it is observed that a small amount of white apatite-like particles are formed on the surface of titanium, which indicates the low bioactivity of the titanium substrate. A slight increase in apatite growth is seen at the level of titanium oxide nanotubes compared to titanium. In the case of coated samples, it is observed that the surface of the samples is covered by a new layer of apatite. Increasing the intensity of Ca and P peaks in the coated samples indicates the formation of an apatite layer on the surface of the coated samples. As the concentration of CNT in the coating increases, the preferred sites for bone-like apatite germination have increased. In composite coatings containing CNTs, CNTs with carboxyl functional group are suitable places for HA germination [18]. Functionalized CNTs have negatively charged carboxyl groups, adsorb calcium ions and SBF-soluble phosphate and form a new apatite layer.

#### Assessment of cell adhesion and cytotoxicity

**Fig. 9:**
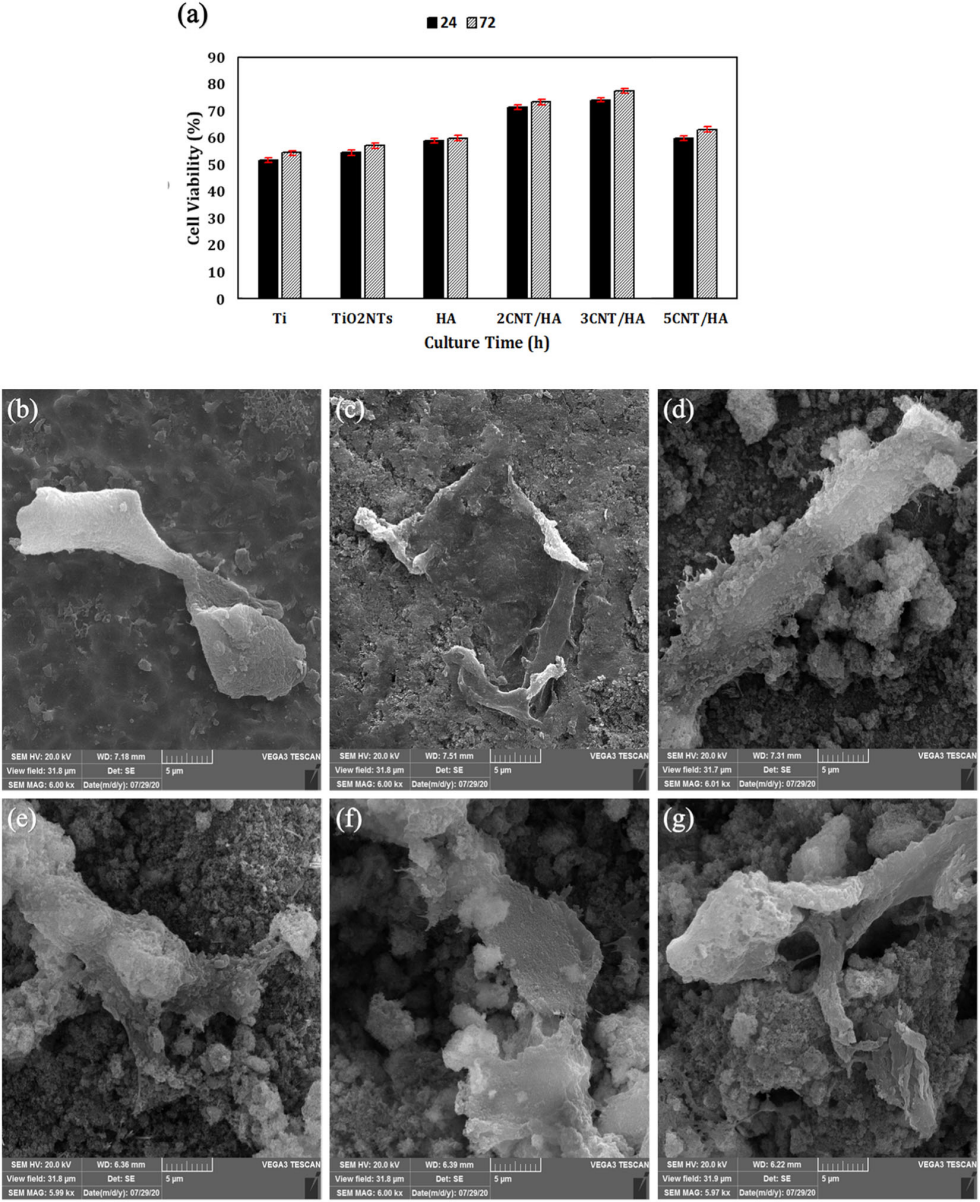
**a** Cell viability of uncoated and coated titanium samples at 24 and 72 h. SEM images of the morphology of MG63 cells on the surface of samples: (**b**) titanium, (**c**) anodized titanium, (**d**) HA, (**e**) 2CNT/HA, (**f**) 3CNT/HA and (**g**) 5CNT/HA coatings

The results of the MTT test are shown in Fig. 9a. All coated samples showed better biocompatibility than titanium sample, which can be attributed to the porous surface morphology, surface roughness and high hydrophilicity.

The results in Fig. 9a show that during the culture time from 24 to 72 h, cell viability increased for all samples except 5CNT/HA composite coating. The decrease in cell viability in the 5CNT/HA sample is probably due to the increase concentration of CNTs particles in this coating, which has led to agglomeration of the CNTs [47]. The role of agglomeration of carbon nanotubes in their toxicity is not yet fully understood. Wick et al. [48]. Showed that the CNT-agglomerates showed the highest toxicity compared to dispersed CNTs, and after three days of incubation in cell cultures, the CNT-agglomerates induced round-shaped cell morphology of the MSTO-211H cells. Mutlu et al. [49]. Concluded that the toxicity of SWCNTs was mainly related to aggregates. They found that dispersed SWCNTs were taken up by alveolar macrophages through mechanisms that gradually cleared over time, such as cilia via mucosal clearance; While aggregated SWCNTs showed a granulomatous structure with mild fibrosis in the mouse trachea.

The titanium and anodized titanium showed 51.8% and 54.5% viability after 24 h of culture, respectively. Also, composite coatings resulted in better cell proliferation compared to other samples. The highest cell viability was obtained for 3CNT/HA samples with 73.2% and 74.7% for 24 and 72 h of culture, respectively. Studies on the biocompatibility of HA-CNTs coatings by other researchers also indicate that CNTs in HA coatings enhances osteoblast proliferation [14, 41, 50]. Factors that may increase cell proliferation and growth at the HA-CNT composite surface include: (1) Binding of proteins on the CNTs surface from the culture medium [51]; (2) the porous structure, high specific surface area and surface energy of CNTs [52]; (3) Higher porosity content in HA-CNTs composite coatings [53]; (4) The bioactive nature of CNTs, Cell viability in CNTs coatings has increased compared to other coatings [54].

Figure 9b shows the adhesion and expansion of MG63 cells on the surface of uncoated and coated titanium. According to SEM images, the proliferation of osteoblast cells on the smooth surface of the substrate is relatively weak, while the anodized titanium surface improves cell adhesion and flattening. Nano surfaces provide reactive surfaces and more sites for protein absorption. As can be seen from Fig. 9d–g, along with the MTT test results, adhesion and dilation of MG63 cells are seen on the surface of samples of HA-CNTs coating samples.

## Conclusions

HA-CNTs composite coatings were successfully electrophoretically deposited on an anodized titanium surface at 10 volts for 8 min. The following observations were made.

1. Increasing the concentration of CNTs from 0 to 5 wt.% led to improved surface uniformity and reduced cracks in the coating. A HA-CNTs composite coating with an approximate thickness of 10 microns was obtained.
2. The adhesion of the coatings to the substrate increased with increasing concentration of CNTs and the coating with a concentration of 5 wt.% had the highest adhesion to the substrate.
3. The highest cell viability was obtained in the coating with a concentration of 3 wt.% of CNTs with a value of 74.7% after 72 h of culture.
